# Primary rhinoplasty: An Indian perspective

**Published:** 2008-10

**Authors:** Uday Bhat, Bharat Patel

**Affiliations:** Department of Plastic Surgery, Topiwala National Medical College and BYL Nair Charitable Hospital, Mumbai, India

**Keywords:** Rhinoplasty, cartilage grafts

## Abstract

The spectrum of indications for rhinoplasty in Indian patients is very wide. An ill-defined nose with lack of projection and broad osteocartilagenous framework is the commonest problem. A large number of patients require narrowing of the framework by osteotomies, alar wedge resection, and augmentation by a suitable fill, preferably a cartilage graft. The technique of rhinoplasty in Indian patients with special emphasis on cartilage grafting has been discussed here. Cartilage grafts, when used as a fill, produce soft, natural results.

## INTRODUCTION

Rhinoplasty is perhaps one of the most fascinating of all aesthetic procedures. Not only does it require orientation in three dimensions, but the surgeon also has to develop a so-called “feel” as the exposure is limited. Additionally it is an operation of judgment rather than technique and has a long learning curve. In this era of aesthetic surgery, proficiency in primary rhinoplasty becomes an essential requirement for modern day plastic surgeons. Literature is often divided as to the best technique,[1] but emphasis should be laid on the individuality of the case and familiarity with different manoeuvers.

In the current article, the spectrum of patients seeking rhinoplasty at our institute, the corrective operations, the technique of cartilage grafting and the results are discussed.

## MATERIALS AND METHODS

In the last 15 years, 316 patients were operated for primary rhinoplasty at our institute. 192 were females and 124 were males. Age varied between 17 to 44 yrs, as shown below

**Table:** 

	17-25 yrs	26-35 yrs	> 35 yrs
Female	117	71	4
Male	85	39	0

The indications were classified according to the primary deformity or the dominant feature: [Table 1].

**Table 1:** Indications with their numbers

*Indication (dominant deformity)*	*Number of patients*
Ill-defined (typical) nose	148
Lack of projection (augmentation)	34
Contour deformities	21
Deviated nose	39
Dorsal hump	10
Small (foreshortened) nose	4
Long nose (drooping tip)	15
Tip deformities	11
Cleft lip nose	36

1. Ill-defined nose: A nose that lacks projection, has broad osteocartilagenous frame work (OCF), has a broad lobule [Figure 1]. This variety constituted nearly 40% of all our patients. Such noses are encountered very often in clinical practice, hence this category is generally referred to as a ‘typical nose’. These noses classically require augmentation, narrowing of the pyramid by osteotomies, tip plasty and alar wedge resection (AWR).

**Figure 1:**
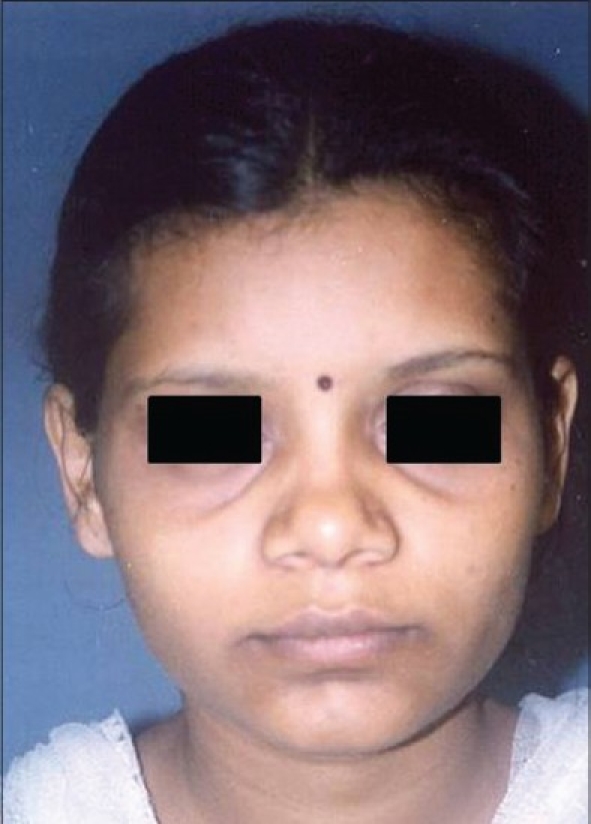
A typical Indian nose. The nose lacks projection, the osteocartilagenous framework is broad, the tip is ill-defined and the lobule is also broad
2. Lack of projection of osteocartilagenous framework (OCF): The OCF is not broad; hence the nose does not require osteotomies. Augmentation may be done by cartilage graft / bone graft/ implant. AWR / tip plasty only if required.
3. Contour deformities: The nose has adequate overall projection. Contour defects include lateral depressions, dorsal deformities (post traumatic or post SMR supratip depressions, nasofrontal depressions). These are corrected by cartilage grafts.
4. Deviated nose: Generally there is deviation of the septum. The septum has to be corrected by septoplasty. High deviations involving the bone may require ethmoid osteotomy [Figure 8 f]. Lateral and medial osteotomies are required for correction of the lateral walls. Residual deformity, if any, is camouflaged by cartilage grafts. Very few mild deformities (without septal deviation) can be corrected by camouflage alone.

**Figure 8f:**
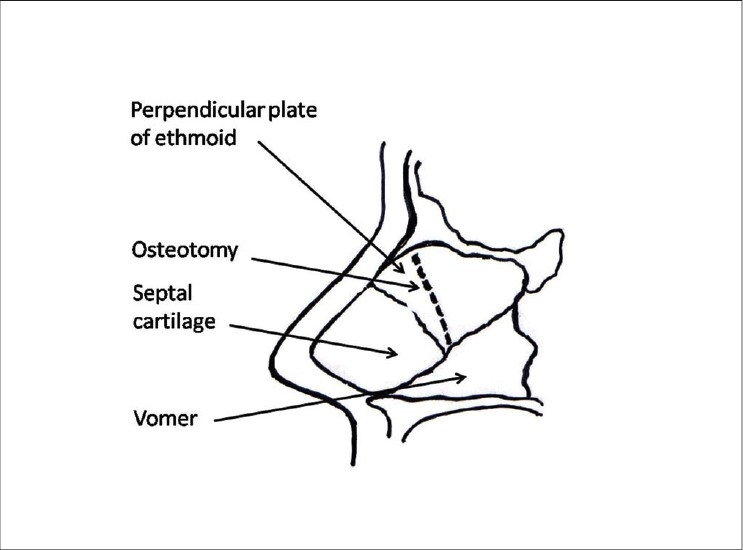
The ethmoid osteotomy
5. Dorsal hump (those associated with deviated nose are included in deviated nose): Dealt with by hump removal and osteotomies.
6. Small or foreshortened nose: Corrected by cartilage graft / bone graft.
7. Long nose (drooping tip): Corrected by cephalic trim, excision of caudal septum.
8. Tip deformities: Inadequate projection, boxy tip etc.
9. Cleft lip nose: Mere correction of cleft deformity alone may not be enough to obtain a satisfactory aesthetic result. Additional procedures like augmentation, osteotomies and alar wedge resection may be required. Hence, these operations should be called ‘rhinoplasty’ rather than ‘correction of cleft lip nose’. Most of the cases require an open technique.[2] Closed technique[3,4] can be used for mild deformities.

Planning: All the operations were planned by clinical examination, careful analysis of photographs and tracings of the profiles. For cases of augmentation and contour fill, a template of the defect was made. This was used as reference to shape the graft [Figure 2a, b]

**Figure 2a:**
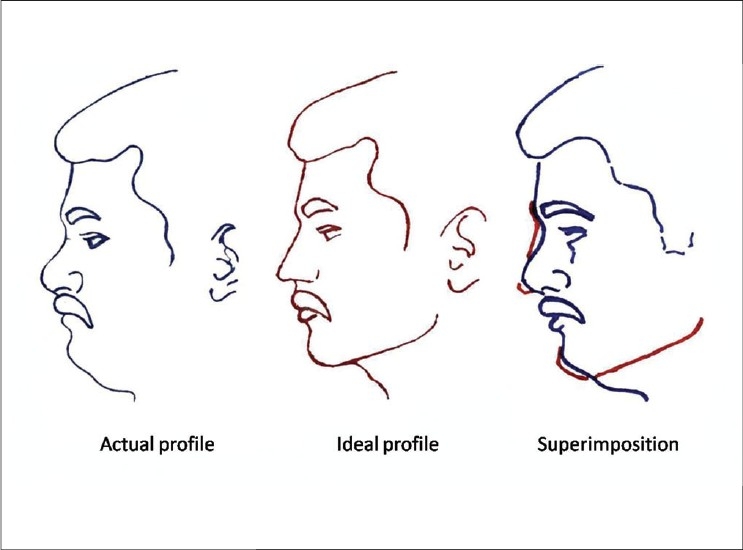
The superimposition of actual and an imaginary ideal profile (based on the surgeon's judgment and patient's expectations). The areas that require either augmentation or reduction can thus be identified

**Figure 2b:**
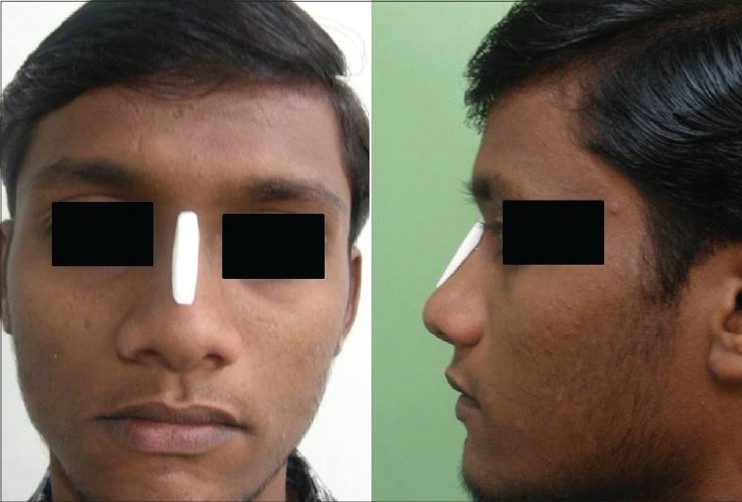
Carving a soap template. The template is used as a reference to shape the cartilage graft

## OPERATIVE TECHNIQUE

### A] Exposure

A Closed approach was used for most of the cases.[5–7]

Intercartilagenous incision + rim incision (bipedicled flap): For Ill-defined (typical) nose.

Infracartilagenous incision only (rim): For augmentation, contour deformities, tip graft, small nose.

Transfixion incision: Mainly used for deviated nose, hump, long nose.

Open approach:[8,9] Cleft lip nose, tip deformities.

The open approach seems attractive as it gives excellent exposure. Accurate closure of this incision is required. Improper closure is one of the causes of distortion of lobule.

### B] Technique of different manoeuvers

1. Osteotomies [Figure 8]: Lateral and transverse osteotomies[2,10] were done by external / percutaneous technique with a 2 mm osteotome; medial osteotomy with a 5 mm straight osteotome with a guard (described later)
  Ethmoid (perpendicular plate) osteotomy[2] was done by internal technique with a 5 mm osteotome without guard.
2. Cartilage grafting: Most of the augmentations and contour fills were done by cartilage grafting.
  - Stacked cartilage graft:[2,11] Conchal cartilage was harvested from one or both the ears and septal cartilage grafts as necessary [Figure 3a]. It was straightened by cross hatching on the concave side and cut into strips [Figure 3b]. These were stacked together with additional strips of septal cartilage as top layer.[1] The several layered graft was shaped to resemble the previously made template [Figure 3c]. Grafts of various sizes and shapes can be fabricated as shown [Figure 3d]. These were inserted in a generous pocket and maintained in position by pull out sutures of 6-0 nylon[12] [Figure 4].

**Figure 3a:**
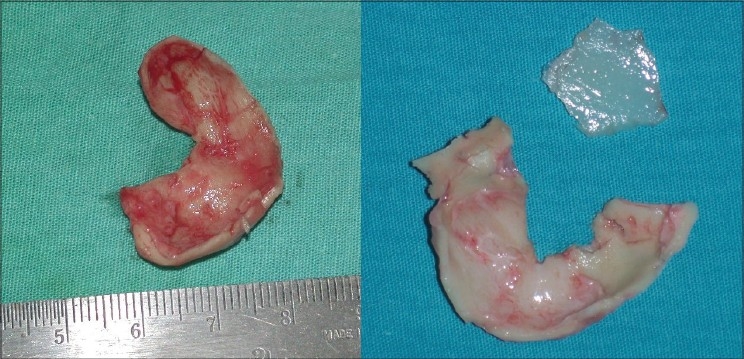
The harvested conchal and septal cartilages

**Figure 3b:**
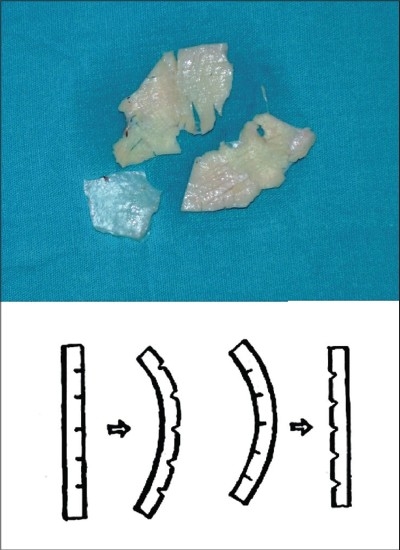
Straightening of the cartilages by scoring incisions

**Figure 3c:**
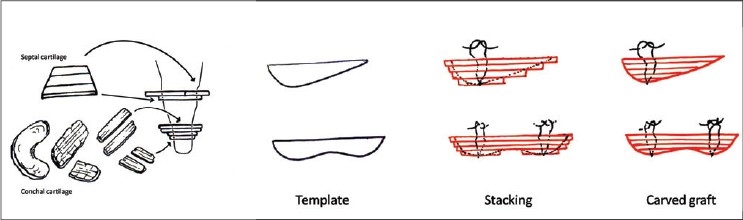
The cartilages are cut into multiple strips of approximately 3 to 5 mm width. A multilayered stack is prepared with conchal cartilage strips at the bottom and septal cartilage strips on top. Multiple 6-0 nylon sutures are required to hold the pieces in place. A graft of desired shape is carved, using the soap template as a reference guide

**Figure 3d:**
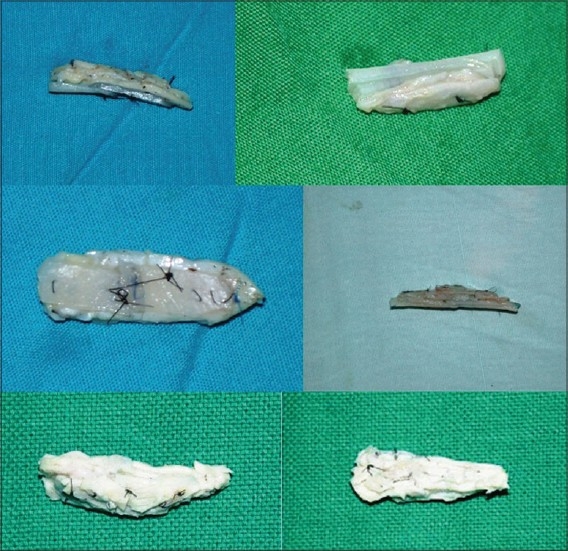
Stacked grafts of various sizes and shapes, ready for insertion. The two grafts at the bottom are made from conchal cartilage alone, as the septal cartilage was not available because of previous submucous resection

**Figure 4:**
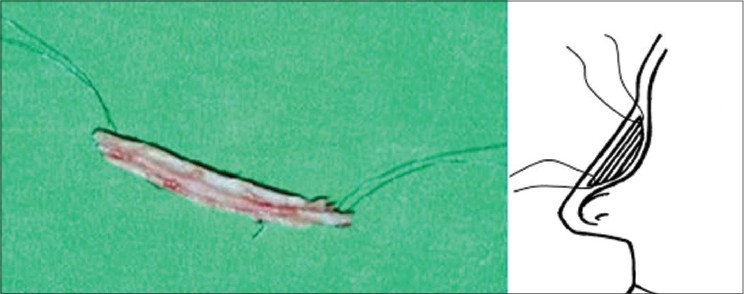
Two threads of 6-0 nylon at either end, brought out as pull out sutures using a straight needle, hold the graft in place
  - Costal cartilage / costochondral graft: 9th or 10th costal cartilage was harvested and straightened using Gibson's principle of balanced cross section[13] (Controlled scoring on the concave side of the curvature) [Figure 5a]. Costal cartilage provides abundant volume and can be used where stacked conchal grafts would be inadequate. These are used mainly in cleft lip noses for augmentation of the dorsum and alar base and for the columellar strut. Costochondral graft is used for cantilever effect by fixing over bony dorsum[14] [Figure 5b].

**Figure 5a:**
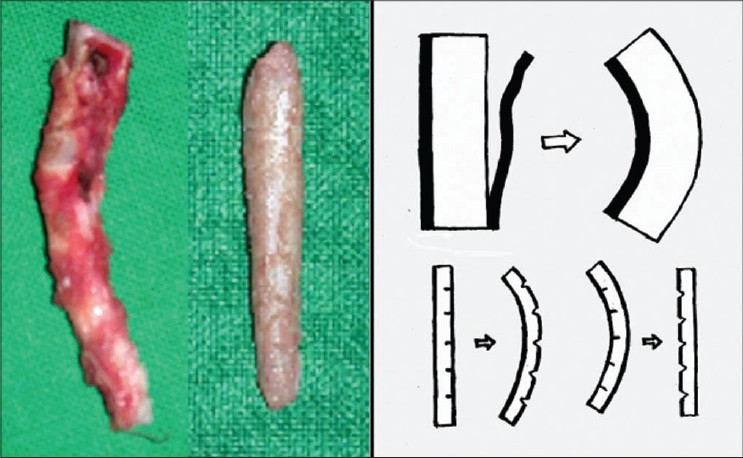
Straightening of costal cartilage by scoring incisions on the concavities. It is better to wait for half an hour before insertion, as most of the warping takes place by that time

**Figure 5b:**
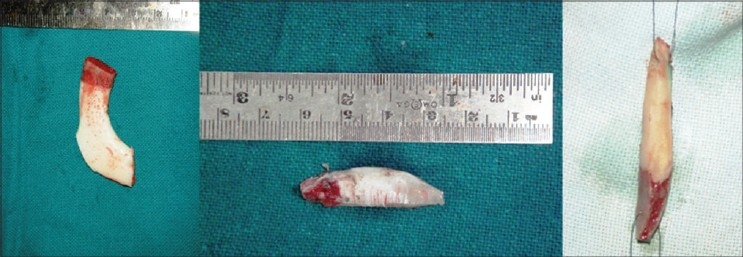
Two examples of costocondral graft. The proximal bony part can be used for screw fixation
3. Tip plasty: Done by modified Lipsett technique.[2] The cartilages were delivered as bipedicled flaps. The excess cephalic portion was excised. The domal area of the remaining portion was weakened by scoring or cross hatching [Figure 7].

**Figure 7:**
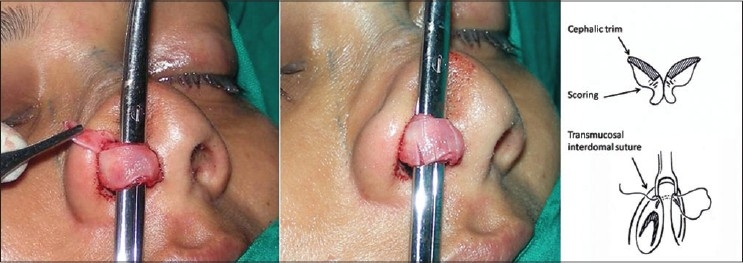
The delivery technique for tip plasty. The cephalic portion of the alar cartilages is excised and the domes are weakened by scoring incisions. The interdomal suture is taken at the time of closure
4. Alar wedge resection (AWR): done by modified Weir technique[2,15] [Figure 9].

**Figure 9:**
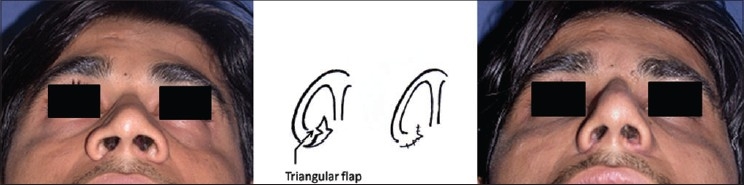
Alar wedge resection. A small triangular flap at the sill preserves the natural contour of the nostril

The technique of the most commonly performed operation - correction of ill defined or ‘typical nose’ is described in greater detail here.

### Technique of rhinoplasty for typical nose

Examination of the nose and analysis of the photographs is done as usual.

Tracings of the profile [Figure 2a]: Many patients are unaware how they look in profile. A patient's perception of his face is the familiar frontal view and that too as a mirror image. It is a good practice to make a tracing of the profile and superimpose it with a drawing of an imaginary ideal profile. This gives an idea of the areas where the nose requires a fill and the areas where the tissues are in excess.

Carving of templates [Figure 2b]: Templates can be carved out of a cake of soap. These can be used as a reference to shape a graft or implant.

Filling the defect with clay also gives a reasonable idea of the volume and shape of the fill.

The basic technique is generally described as stages or steps. It is better to call them components as the sequence of execution is not rigid. In fact the stage or component of hump reduction is not even required in eighty percent of the cases.

Western literature mentions the use of local anaesthesia for rhinoplasty. In reality, very few operations are possible under local anaesthesia. (This is partly due to prohibition of cocaine use in India).

The marking would include defining the midline, areas that need augmentation or reduction, the osteotomy lines, lines for AWR etc.

The conchal cartilage (costal when planned) is then first harvested and one surgeon contours and shapes it according to the needs [Figure 3c, d]. The other surgeon proceeds with the nose.

For a typical nose, the best exposure would be to use both transfixion and infracartilagenous (rim) incisions and deliver the alar cartilages as bipedicled flaps. If AWR is planned, taking these incisions beforehand improves the overall exposure.

Degloving of the skin and soft tissue envelope (SSTE) should be done in a plane close to the osteocartilagenous frame work. An easy way is to expose the nearest cartilage (lateral for intercartilagenous, alar for rim) and follow the surface upwards. Extensive degloving is required in cases where the envelope needs to be draped on a significantly modified framework, as in major augmentation.

The cartilagenous septum is exposed by elevating the mucoperiochondrial flaps bilaterally. Raising these flaps in a correct bloodless plane is important. Near the caudal end, the cartilage is scratched with a knife till a pale white surface is exposed. This plane is followed to raise the flaps. The dorsal border of the septum is separated from the attachment of lateral cartilages by two parallel incisions on either side [Figure 6 a]. The upper ends of these incisions will lead the surgeon to the beginning points of the medial osteotomies [Figure 6 b]. The septal graft is harvested next, keeping the dorsal and caudal borders (the L- strut) intact and handed over to the first surgeon, who would then add the septal strips to the conchal strips to prepare graft/s of desired shape [Figure 3c, d and 4].

**Figure 6a:**
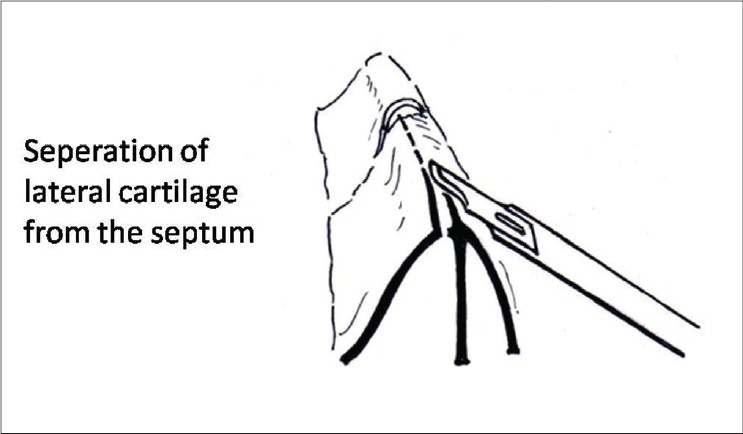
An incision to separate the lateral cartilages from the septum

**Figure 6b:**
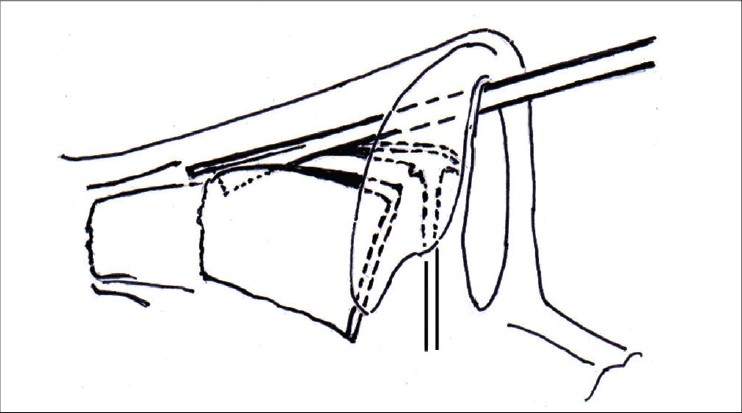
The proximal end of this incision is the beginning point of medial osteotomy

Tip plasty is performed by delivery of the alar cartilages, excision of the excessive cephalic portion (‘the cephalic trim’), excision of excessive interdomal tissue and scoring or weakening the domes [Figure 7]. The transmucosal interdomal suture is taken later, at the time of closure [Figure 7].

The lateral osteotomies [Figure 8 a,b] should preferably be low to low and should stop at the level of the medial canthi superiorly. The nose should not be narrowed above this level as it breaks the diverging curved aesthetic lines. Lateral osteotomy is possible through a single stab incision and maneuvering the 2 mm osteotome above and below, along the previously marked line. A transverse component should then be added, through another horizontal stab incision at the canthal level. The transverse osteotomy breaks the bones in a controlled manner compared to a manual greenstick out-fracture. The medial osteotomies [Figure 8 c,d], done with a 5 mm osteotome with guard, should separate the nasal bones from the perpendicular plate of the ethmoid. The nasal walls are then shifted medially with gentle compression applied over a wet gauze [Figure 8 e]. The ethmoid osteotomy is not indicated in a typical nose. However, when required to correct the high bony deviation, it is done by a 5mm osteotome placed between the perpendicular plate and the mucoperiosteal flap on the side of deviation [Figure 8 f].

**Figure 8a,b:**
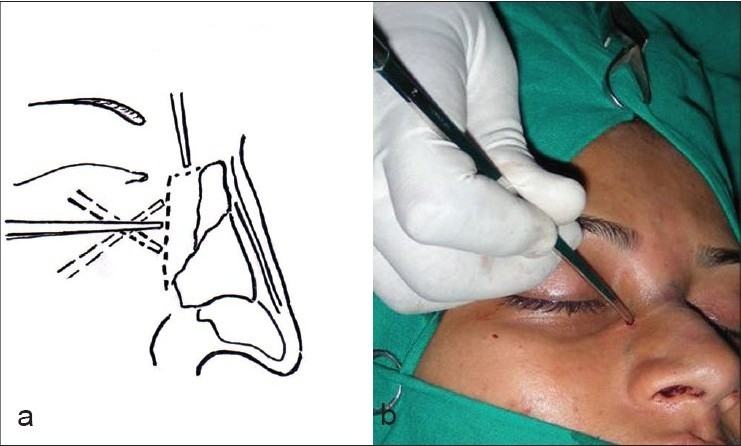
The percutaneous lateral and transverse osteotomies

**Figure 8c,d:**
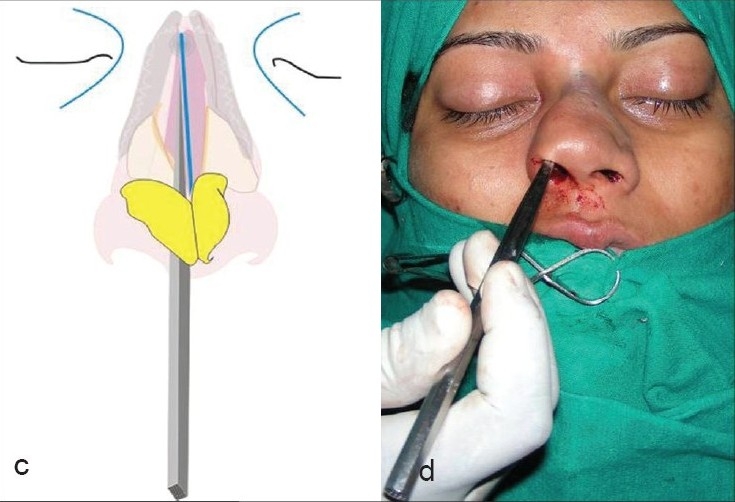
The medial osteotomy

**Figure 8e:**
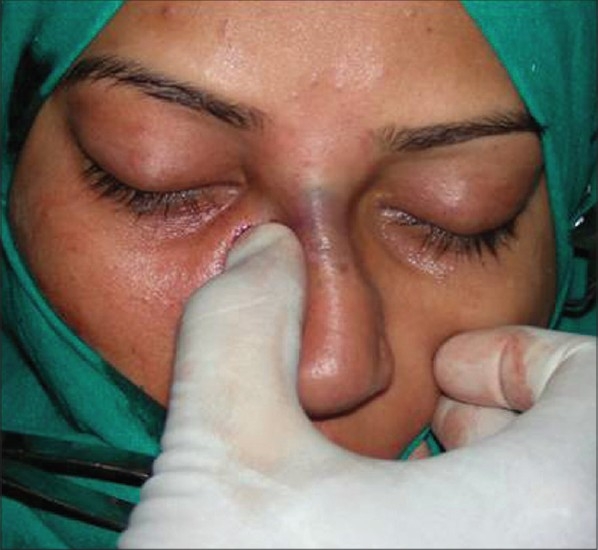
Shifting the lateral nasal walls medially

In cases of hump removal, the osteotomies are designed to close the open roof, i.e. to bring the upper portion of nasal walls towards the midline. In a typical nose, the purpose of these osteotomies is to shift the lower portion of the nasal walls medially so as to narrow the base of the pyramid [Figure 8 g].

**Figure 8g:**
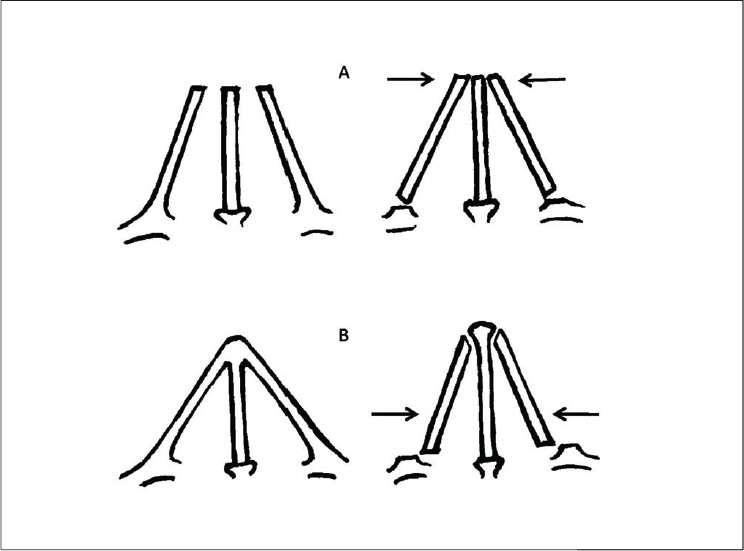
A - The osteotomies serve to close the ‘open roof’ in cases of hump reduction, by shifting the upper portion of the nasal walls medially. B – The purpose of osteotomies in a typical nose is to bring the lower portion of the nasal walls medially, so as to narrow the framework

The stacked graft is inserted at the desired place. The two threads at either ends are brought out as pull out sutures, using a straight needle and the ends are kept long [Figure 4].

The Alar Wedge Resection is done by modified Weir technique. Excision is designed so as to narrow the nostril sill. A small triangular flap breaking the straight incision lines preserves the natural contour of the nostrils [Figure 9]. The transmucosal interdomal suture is now taken [Figure 7], followed by closure of incisions. The pull out sutures are tied last, over a bolster of tulle gras (to be removed at day 5). The shape of the nose is maintained with porous adhesive tape. Plaster of Paris splint is kept for three weeks to maintain the nasal walls at the new medial position.

## RESULTS

Enumerated below are the details regarding the exposure and various manoeuvres performed [Table 2].

**Table 2:** Exposure type and manoeuvres

*Indication (dominant deformity)*	*Exposure*		*Manoeuvers*		
		
	*Closed*	*Open*	*Osteotomies*	*AWR*	*Fill*
Ill-defined (typical) nose	139	9	148	148	148
Lack of projection (augmentation)	29	5	-	11	34
Contour deformities	21	-	4	2	21
Deviated nose	31	8	36	4	15
Dorsal hump	10	-	10	2	1
Small (foreshortened) nose	4	-	-	-	4
Long nose (drooping tip)	12	3	6	1	4
Tip deformities	4	5		2	6
Cleft lip nose	8	28	15	8	36
	258	58	219	176	269

Most of the operations were possible by the closed approach. Open approach was required mainly for primary tip deformities and cleft lip noses. The most frequently performed manoeuvers were cartilage grafting, osteotomies and alar wedge resection.

Type of fill used is described in Table 3.

**Table 3:** Type of filler for correction

*Indication (dominant deformity)*	*Type of fill*				
	
	*Stack*	*Turkish delight*	*Costal/ costochondral*	*Bone (iliac)*	*Implant (polyethylene)*
Ill-defined (typical) nose	146	1	1	-	-
Lack of projection (augmentation)	17	1	3	9	4
Contour deformities	17	-	4		
Deviated nose	15				
Hump	1				
Small (foreshortened) nose	2		2		
Long nose (drooping tip)	4				
Tip deformities	6				
Cleft lip nose			36		
	208	2	46	9	4

38 patients received grafts at more than one site.

Overall results were classified according to patient satisfaction, satisfaction of the surgeon and assessment by a neutral observer (usually an ENT / Surgical colleague) [Table 4].

**Table 4:** Results

*Results*	*No. of patients*
Excellent	77
Good	178
Average	53
Poor	8

Most excellent and good results were seen in cases of ill-defined nose, augmentation and deviated nose (Representative patients 1,2,4). Stacked cartilage grafts performed very well. They produced a soft, natural result. Warping, lack of volume and resorption were not a problem. The largest stacked graft was a 10 layered graft of conchal (bilateral) and septal cartilages; measuring 10 mm in thickness and 25 mm in length. The Turkish delight technique did not work well in the authors' hands.[16]

Average results mostly belonged to the categories of cleft lip nose and small nose. Poor results were due to two instances of complete resorption and two instances of partial resorption of iliac bone graft, infection and partial resorption of cartilage graft in one case, and failure to achieve good symmetry in three cases of cleft lip nose.

Almost all cases of deviated nose required septal correction and osteotomies. Correction by camouflage alone was possible only in three cases having no significant septal deviation.

### Complications of the fill

Cartilage-

Warping – 1 (costal), partial resorption- 1, shift of position- 1, extrusion- nil, donor site morbidity- nil

Bone (iliac crest)-

Complete resorption- 2, partial resorption- 2, donor site morbidity (pain/difficulty in walking in post op period)- 3

Implant (polyethylene): nil

### Representative cases

**Figure 10:**
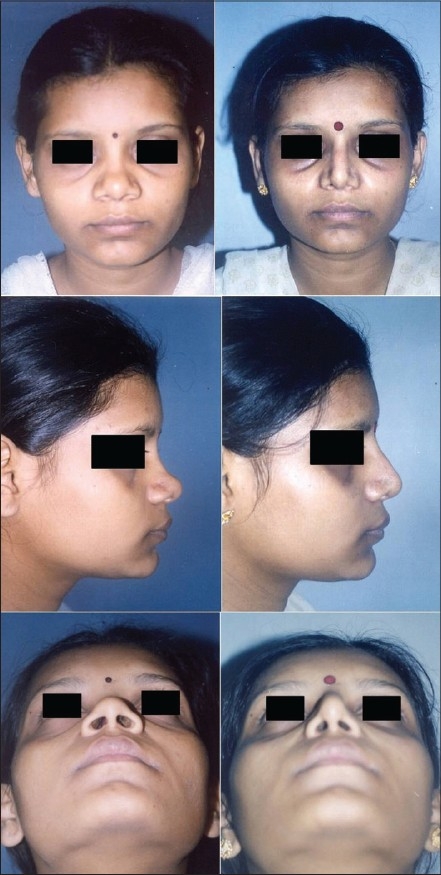
(Patient 1) Typical or ill-defined nose. Osteotomies and a seven layered cartilage graft have produced the two dorsum defining lines in the frontal view. Augmentation of the profile is significant and tip has better definition

Patient 1 [Figure 10]: Ill-defined or typical nose. This 20 year old girl had inadequate projection, broad OCF, broad lobule and broad tip. This was corrected by osteotomies, tip plasty, AWR and onlay graft of seven layers.

**Figure 11:**
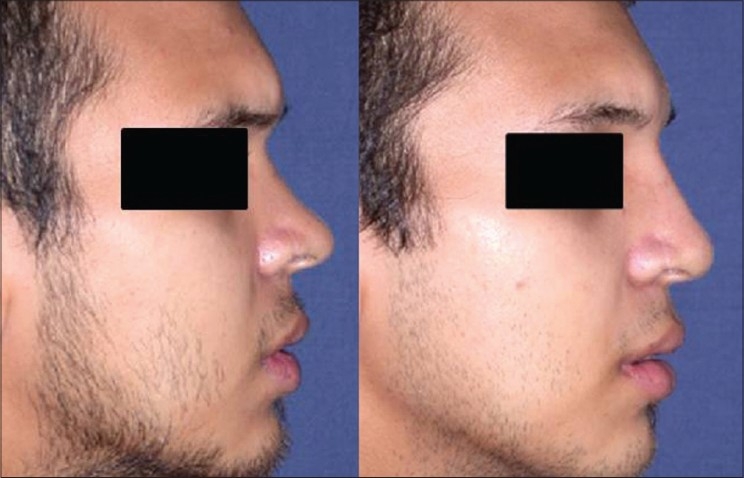
(patient 2) Augmentation by a stacked septal cartilage graft

Patient 2 [Figure 11]: Lack of projection. Augmentation was done by a three layered septal cartilage graft.

**Figure 12:**
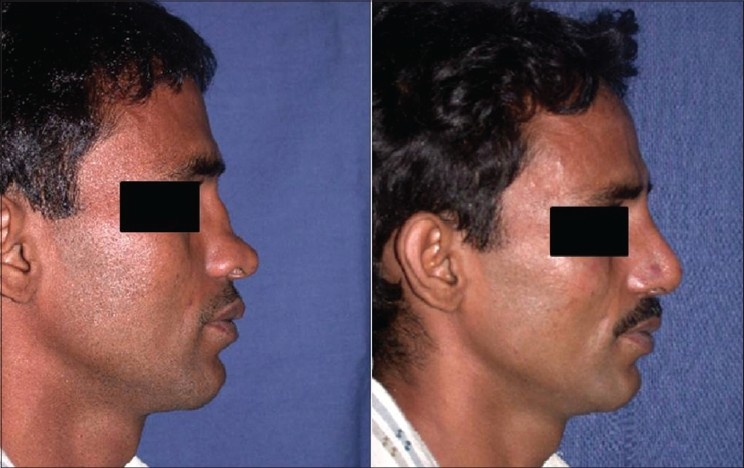
(patient 3) Previous submucous resection has resulted in a supratip depression. A simple onlay graft to the dorsum may sink because of lack of structural support. The deformity is corrected by a cantilevered costochondral graft

Patient 3 [Figure 12]: Supratip contour deformity and dorsal collapse following a submucous resection of septum (SMR). There was lack of structural support, due to removal of a large portion of septal cartilage. Correction was done by a cantilevered costochondral graft.

**Figure 13:**
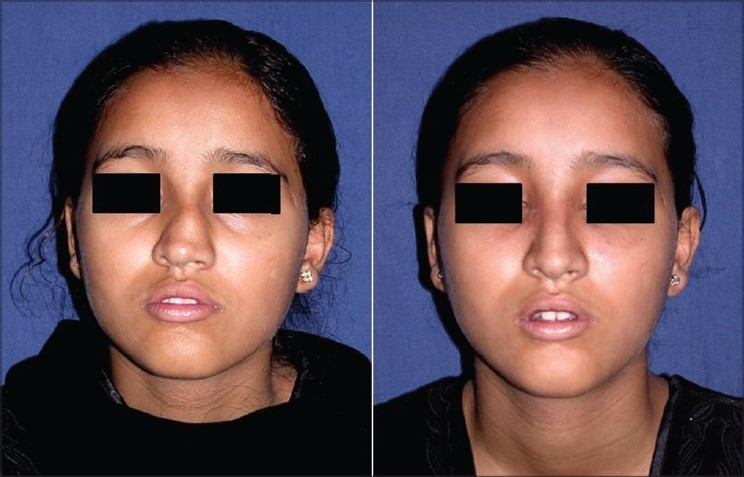
(patient 4) Deviated nose. Total correction of the septum was achieved by straightening the septal cartilage by scoring incisions and ethmoid osteotomy to correct the high bony deviation. Lateral and medial osteotomies and a small conchal graft to fill the supratip area were also required

Patient 4 [Figure 13]: This 18 year old girl had trauma to the nose in childhood resulting in a deviated nose. The septum was deviated to the right and also buckled, resulting in a supratip depression. Apart from lateral and medial osteotomy, the perpendicular plate of ethmoid was broken to correct the high bony deviation. Supratip depression was filled by conchal cartilage graft.

**Figure 14:**
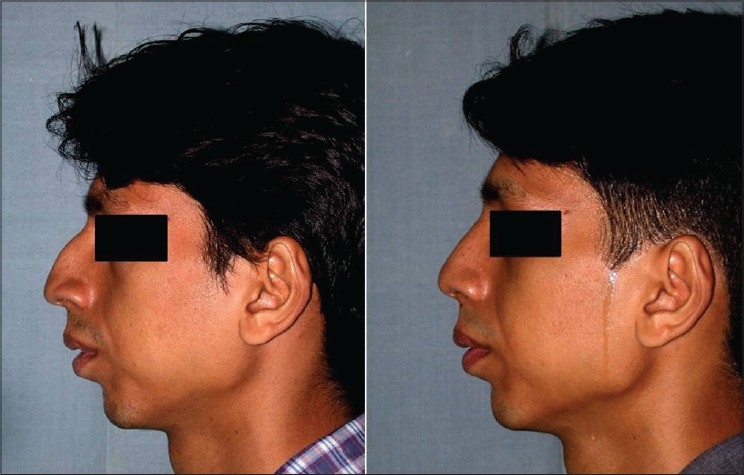
(patient 5) Conservative removal of the dorsal hump

Patient 5 [Figure 14] Dorsal hump: Conservative hump removal was done to maintain a convex profile line. Excessive removal may result in a straight dorsum, breaking the natural profile line.

**Figure 15:**
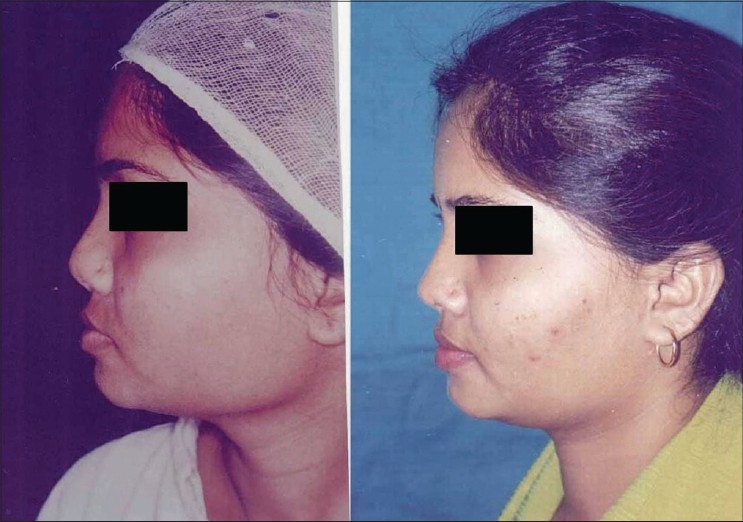
(patient 6) Correction of short nose by a four layered conchal cartilage graft

Patient 6 [Figure 15]: This was 22 yr old girl with foreshortened nose. Lengthening was done by a four layered conchal cartilage graft.

**Figure 16:**
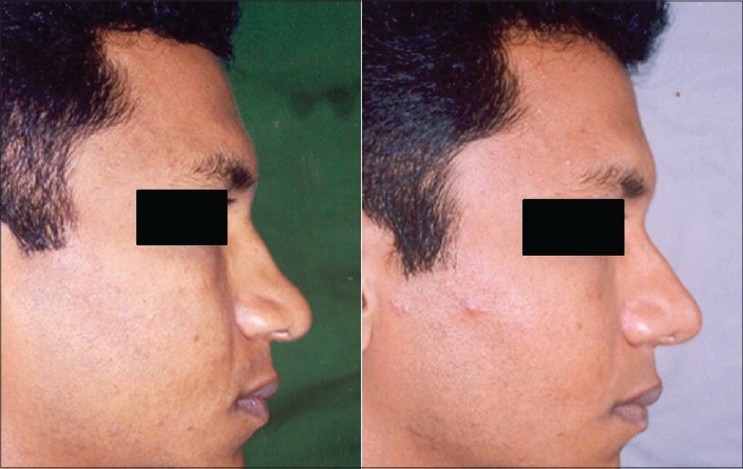
(patient 7) A long nose corrected by cephalic trim and excision of 3 mm of caudal part of the septum. The dorsum is augmented by a cartilage graft

Patient 7 [Figure 16]: This patient had a long nose which was shortened by cephalic trim of the alar cartilage and excision of 3 mm of caudal part of the septum. Augmentation of the dorsum was done by two layered septal graft.

**Figure 17:**
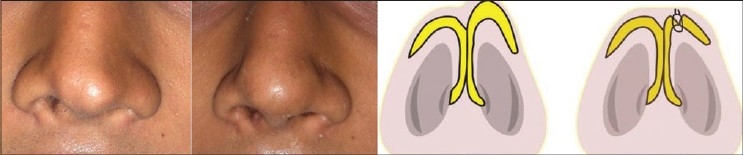
(patient 8) Excessive projection of alar cartilage corrected by excision of the dome

Patient 8 [Figure 17]: This patient had a tip deformity because of too much projection of left dome. The excess domal portion of the cartilage was excised, and continuity was established by suturing the lateral and medial part.

**Figure 18:**
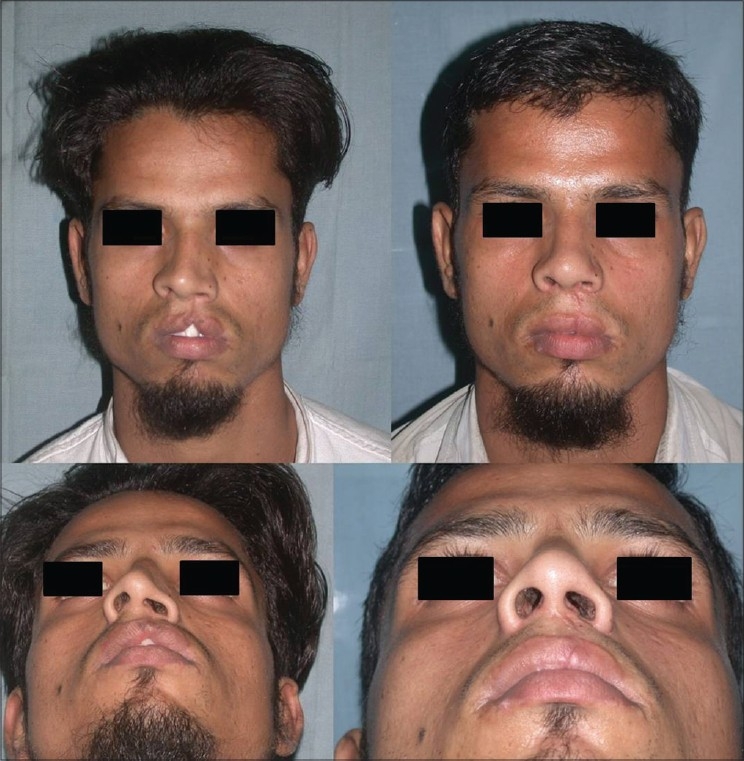
(patient 9) Cleft lip nose. Septal correction, osteotomies and cartilage grafts to the left dome and to the alar platform were required for correction

Patient 9 [Figure 18]: The cleft lip nose was seen in this patient: The columella is short, right dome is collapsed, and there is retrusion of the alar platform. By open approach, the columella was lengthened and domal symmetry was achieved by repositioning of the alar cartilages and a graft to the right dome. The alar platform and nasal dorsum were augmented by costal cartilage graft.

**Figure 19:**
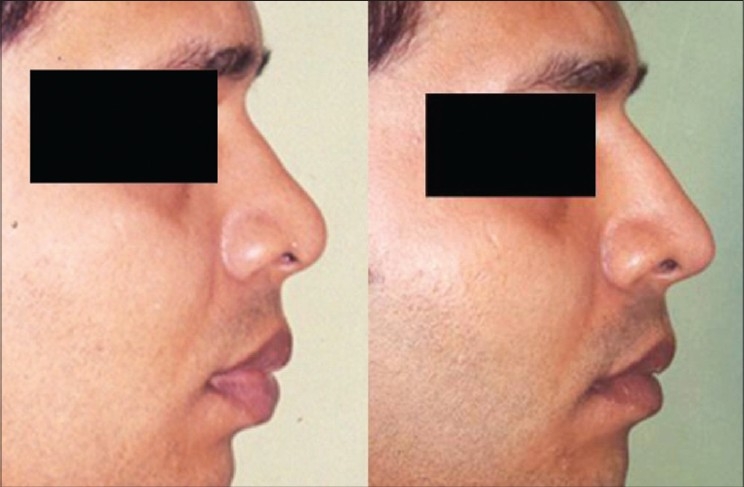
(patient 10) An ill-defined tip corrected by conchal cartilage graft. The dorsum is also augmented by a single layered septal graft

Patient 10 [Figure 19]: An ill defined tip was corrected by two layered tip graft.

**Figure 20:**
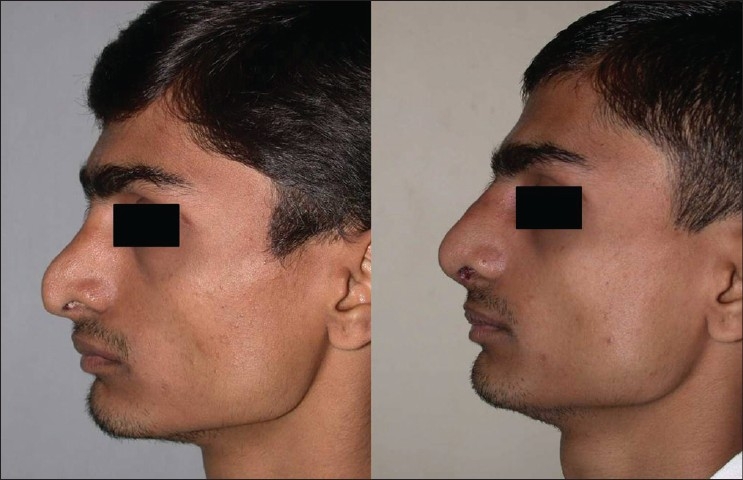
(patient 11) Parakeet nose. There is excessive projection of the cartilaginous (lower) part of the septum. The projecting dorsal border of septum and lateral cartilages is excised and upper part of the dorsum is augmented with a cartilage graft

Patient 11 [Figure 20]: This patient had a contour deformity (Parakeet nose) which was corrected by lowering of lower part of septum and augmentation of the upper part.

**Figure 21:**
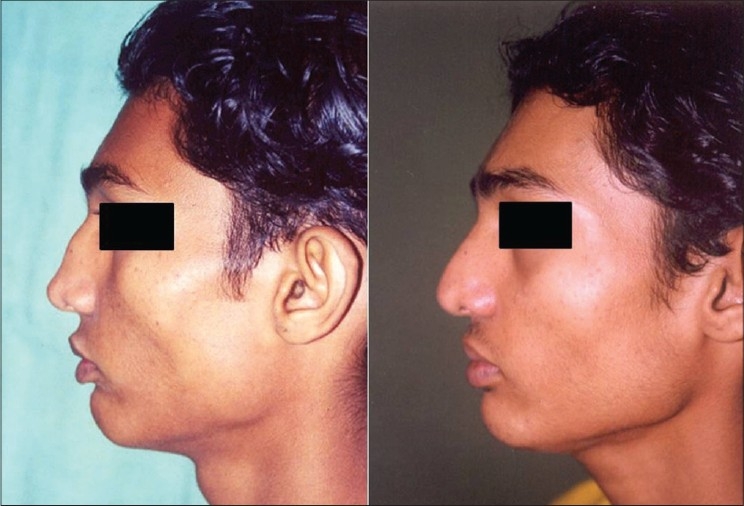
(patient 12) Supratip depression corrected by a three layered conchal cartilage graft

Patient 12 [Figure 21]: Contour deformity in Supratip area in this patient was corrected by conchal cartilage graft.

**Figure 22:**
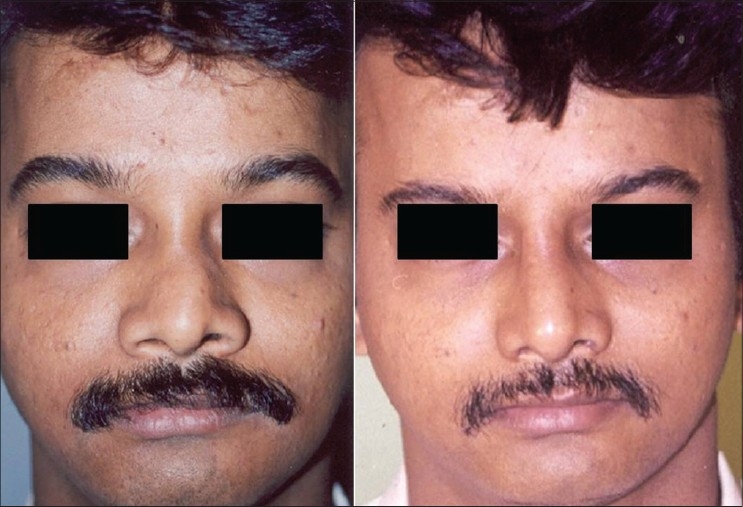
(patient 13) Deviated nose corrected by camouflage alone. This was possible because the septum was not deviated

Patient 13 [Figure 22]: Camouflage was made for a deviated nose by bone graft.

## DISCUSSION

An ill-defined nose is the most commonly encountered problem in clinical practice. These noses typically have a broad OCF, broad lobule and tip and also lack projection. It is easy to think that the broadness is apparent, because of lack of projection. In fact in most cases it is real, and these noses need to be narrowed by osteotomies and AWR. Initially the authors used to follow the closed or intranasal technique for lateral osteotomy, but have now shifted to external percutaneous technique, which gives better control and is easier to perform. It is also better to add a transverse osteotomy at the level of medial canthi, rather than performing a greenstick out-fracture.

The classical reduction rhinoplasty described in western literature[1] may not be fully applied in the Indian context. The typical Indian nose lacks projection and hence requires augmentation more often than reduction. Some noses may even need reduction at one place and augmentation at the other. Hence cartilage grafting is an integral part of Indian rhinoplasty and today's surgeon must be well versed with the technique and use of cartilage grafts.

Stacked cartilage grafts produce soft and natural results.[11] Problems of infection, resorption and warping are almost non-existent. Wrapping these grafts in fascia[2] is not required in Indian patients, as the sharp edges do not show through the thicker skin. In fact, the edges give better definition. Curved conchal cartilage can be straightened by scoring on the concave side. Usually one concha provides enough volume for a graft of 4 to 5 mm in thickness. With both conchae and additional strips of septal cartilage, a large graft, almost a centimetre thick, can be sculpted. Costal cartilage provides the abundant volume required in cleft lip noses. Warping of the costal cartilage can be avoided by preserving the perichondrium and breaking it only along the curvature (the concave side).[13] The only instance of warping in our patient was due to unfamiliarity with technique of scoring and lack of understanding of the Gibson's principle.[13] Although done in only two cases, the Turkish delight technique[16] was a failure at the authors' hands as the grafts could not be properly shaped. Postoperative moulding was not possible because of pain. The authors have no experience of calvarial grafts, but the iliac grafts were disappointing.

## CONCLUSIONS

In Indian context most of the patients seek correction of obvious deformities. The typical deformity is a broad, nonprojecting nose. Cartilage grafting, osteotomies and AWR are important and most frequently performed manoeuvres. In equivocal situations it is prudent to err on the side of putting into practice the important tenets of rhinoplasty – osteotomies, cartilage grafting and alar wedge resection. Good results can be obtained by familiarity with these three techniques.
